# Neuroanatomical association of hypothalamic HSD2-containing neurons with ERα, catecholamines, or oxytocin: implications for feeding?

**DOI:** 10.3389/fnsys.2015.00091

**Published:** 2015-06-15

**Authors:** Maegan L. Askew, Halie D. Muckelrath, Jonathon R. Johnston, Kathleen S. Curtis

**Affiliations:** Department of Pharmacology and Physiology, Oklahoma State University – Center for Health SciencesTulsa, OK, USA

**Keywords:** ventromedial hypothalamic nucleus, arcuate nucleus, lateral hypothalamic area, paraventricular hypothalamic nucleus, sex differences

## Abstract

This study used immunohistochemical methods to investigate the possibility that hypothalamic neurons that contain 11-β-hydroxysteroid dehydrogenase type 2 (HSD2) are involved in the control of feeding by rats via neuroanatomical associations with the α subtype of estrogen receptor (ERα), catecholamines, and/or oxytocin (OT). An aggregate of HSD2-containing neurons is located laterally in the hypothalamus, and the numbers of these neurons were greatly increased by estradiol treatment in ovariectomized (OVX) rats compared to numbers in male rats and in OVX rats that were not given estradiol. However, HSD2-containing neurons were anatomically segregated from ERα-containing neurons in the Ventromedial Hypothalamus and the Arcuate Nucleus. There was an absence of OT-immunolabeled fibers in the area of HSD2-labeled neurons. Taken together, these findings provide no support for direct associations between hypothalamic HSD2 and ERα or OT neurons in the control of feeding. In contrast, there was catecholamine-fiber labeling in the area of HSD2-labeled neurons, and these fibers occasionally were in close apposition to HSD2-labeled neurons. Therefore, we cannot rule out interactions between HSD2 and catecholamines in the control of feeding; however, given the relative sparseness of the appositions, any such interaction would appear to be modest. Thus, these studies do not conclusively identify a neuroanatomical substrate by which HSD2-containing neurons in the hypothalamus may alter feeding, and leave the functional role of hypothalamic HSD2-containing neurons subject to further investigation.

## Introduction

A population of neurons containing 11-β-hydroxysteroid dehydrogenase type 2 (HSD2) have been reported to be located in the ventromedial hypothalamus (VMH; Geerling et al., 2006b). Although the functional role of these neurons is unclear, it is known that HSD2 facilitates binding of aldosterone to mineralocorticoid receptors (MRs; Náray-Fejes-Tóth et al., 1998). In fact, HSD2-containing neurons in the hindbrain nucleus of the solitary tract (NTS) have been implicated in sodium ingestion in response to sodium deficiency (Geerling et al., 2006a; Geerling and Loewy, 2007), which is accompanied by increased aldosterone levels. These observations raise the possibility that HSD2-containing neurons in the VMH also may confer sensitivity to aldosterone and thereby mediate behavioral responses to changes in sodium state. However, alternative possibilities exist. One such possibility is suggested by extensive evidence converging on the role of hypothalamic areas, including the VMH, in the central control of energy balance (Woods and Stricker, 2008). Classic studies in the 1940s and 1950s demonstrated that electrolytic lesions of the VMH cause hyperphagia, increased adiposity, and increased body weight (Hetherington and Ranson, 1940, 1942; Anand and Brobeck, 1951). With more sophisticated experimental approaches, better understanding of the role of the VMH and other hypothalamic areas in the control of feeding and body weight has emerged, particularly in regard to the neuronal phenotypes that mediate these effects.

Interactions among neuropeptide Y (NPY), agouti gene related peptide (AgRP), and proopiomelanocortin (POMC) neurons in the arcuate nucleus (ARC) have been the subject of much investigation (Schwartz et al., 2000; Woods and D’Alessio, 2008), as has the role of the neuropeptide oxytocin (OT; Olson et al., 1991; Verbalis et al., 1993; Uchoa et al., 2009; Olszewski et al., 2010; Sabatier et al., 2013), and catecholamines (Schwartz et al., 2000; Wellman, 2000; Palmiter, 2007). Recently, attention has turned to the role of estradiol and hypothalamic estrogen receptors (ERs) in energy balance. Estradiol is well-known to influence feeding and body weight. Specifically, adult female rats display cyclic changes in feeding that correspond to estrous cycling (Blaustein and Wade, 1976; Eckel et al., 2000). Moreover, removal of endogenous estradiol by ovariectomy promotes hyperphagia and weight gain in rats, both of which are reversed by estradiol treatment (Wade, 1972; Blaustein and Wade, 1976; Roy and Wade, 1977; Geary and Asarian, 1999; Krause et al., 2006). As a steroid hormone, estradiol readily accesses the central nervous system (CNS) and acts at ERs that are broadly distributed throughout the CNS (Simerly et al., 1990; Shughrue et al., 1997; Laflamme et al., 1998). The alpha subtype of ERs (ERα) has been implicated in the control of food intake (Roesch, 2006; Santollo et al., 2007, 2010), and a particularly dense population of ERα exists in the hypothalamic VMH. Indeed, in an interesting parallel to classic VMH lesion studies, Musatov and colleagues used siRNA methods to selectively prevent expression of ERα in the VMH and found that rats over-ate and gained excess weight, while physical activity and metabolic rate decreased (Musatov et al., 2007).

A tantalizing possibility for interactions between estradiol and HSD2-containing neurons in the VMH is suggested by our recent findings that estradiol treatment increased the number of HSD2-containing neurons in the NTS of ovariectomized (OVX) rats (Fan et al., 2010). But is it possible that neuroanatomical associations between HSD2 and estradiol in the VMH are involved in the control of feeding via ERα? If so, several outcomes might be envisioned. First, one might predict co-localization of ERα and HSD2 in VMH neurons. An alternative outcome, based on the ability of estrogens to alter gene transcription (e.g., Marino et al., 2006), would be changes in the numbers of HSD2 neurons, and/or in HSD2 synthesis that parallel changes in estradiol levels. Finally, HSD2 may interact indirectly with other neurotransmitters in the hypothalamus to alter estradiol-mediated effects on food intake. Clearly, these are not mutually exclusive possibilities, nor are they intended to be exhaustive. Rather, given the comparative dearth of information about hypothalamic HSD2-containing neurons, these ideas are intended to serve as a “jumping off” point in exploring the role of HSD2-containing neurons in the hypothalamus. Accordingly, based upon connections among these hypothalamic areas and on our report of increased number of HSD2-containing neurons in the NTS after estradiol treatment (Fan et al., 2010), we began with the hypothesis that interactions between hypothalamic HSD2 and estradiol are important in the control of feeding. This hypothesis, therefore, is the basis of these preliminary studies utilizing immunohistochemical approaches to examine neuroanatomical associations between HSD2 and ERα in the hypothalamus. We also explored the possibility of indirect interactions by examining relationships between hypothalamic HSD2 and OT or catecholamines.

## Methods

### Animals, Surgeries, and Hormone Replacement

Adult female and male Sprague-Dawley rats (Charles River), 90 days of age, were individually housed in plastic cages in a temperature-controlled colony room on a 12:12 light:dark cycle with lights on at 07:00. Rats were adapted to the colony room for a minimum of 7 days before any procedures were initiated and were given *ad libitum* access to Harlan rodent diet (no. 2018) and deionized water throughout the study. All procedures were approved by the Oklahoma State University—Center for Health Sciences Institutional Animal Care and Use Committee.

Female rats were bilaterally OVX under pentobarbital anesthesia (Sigma; 50 mg/kg, ip). After a week of recovery, OVX rats received subcutaneous injections of the oil vehicle (OIL; 0.1 ml; *n* = 6) or 17-β-estradiol-3-benzoate (EB; Fisher; 10 μg in 0.1 ml oil; *n* = 7) on Day 1 and Day 2 of a 4-day replacement protocol. This protocol, based on studies by Woolley and McEwen (1993), has been used extensively in neuroanatomical and behavioral studies (e.g., Woolley and McEwen, 1993; Kisley et al., 1999) including those from our lab (Krause et al., 2006; Fan et al., 2010; Graves et al., 2011).

Male rats (*n* = 5) did not undergo surgery; however, three males received subcutaneous injections of the oil vehicle (0.1 ml) on the same 4-day protocol as OVX rats, while two male rats were untreated. No differences in any measures were observed due to oil treatment of male rats.

### Perfusion, Brain Collection, and Sectioning

OVX and male rats that were given EB or oil injections were weighed on Day 1 and 2, and again on Day 4, after which they were deeply anesthetized with pentobarbital (25 mg/rat). Rats then were perfused transcardially with 0.15 M NaCl followed by 4% paraformaldehyde. Brains were removed, postfixed in 4% paraformaldehyde overnight, and then stored in 30% sucrose at 4°C. Brains were cut into a 1:3 series of 40-μm sections using a cryostat (Leica); sections were stored in a cryoprotectant solution (Watson et al., 1986) at −20°C until processed.

Untreated male rats were weighed only on the day of sacrifice, and then were anesthetized and perfused as described.

### Body Weight and Uterine Weight

Changes in body weight during the 4-day protocol were assessed for OVX and male rats that were given EB or oil injections by calculating the percent change in body weight from Day 4 to Day 1.

After perfusion on Day 4, uterine weights were assessed from a subset of OVX rats (OIL: *n* = 4; EB: *n* = 5). For each rat, the uterus was removed and stripped of fat. A 10-cm segment of one uterine horn beginning at the bifurcation then was cut and weighed.

### Immunolabeling

Free-floating forebrain sections were rinsed in 0.05 M Tris-NaCl, followed by 30 min in 0.5% H_2_O_2_, and then were rinsed in 0.05 M Tris-NaCl before being incubated for 1 h in 10% normal goat serum (NGS) mixed in 0.5% Triton X in 0.05 M Tris-NaCl. One series of sections from subsets of rats then was processed to label markers of various neuronal phenotypes using immunolabeling procedures described below.

Upon completion of immunolabeling, tissue was rinsed in 2% NGS, and then in 0.05 M Tris-NaCl. Sections from each animal were ordered, mounted on gelatin-coated glass microscope slides, and allowed to dry. Slides then were briefly immersed in distilled water, then dehydrated in an ascending series of ethanols (70%, 95%, 100%), and defatted in xylenes before coverslipping with Cytoseal 60 (Fisher Thermo Scientific). Immunolabeling was examined using a Nikon Eclipse 80i epifluorescent microscope equipped with a camera, FITC and rhodamine filters, and NIS Elements software.

#### ERα Immunolabeling

Sections were incubated in the primary antibody (Millipore; rabbit anti-ERα C1355; MM_NF-06-935)^1^ diluted 1:10, 000 in 2% NGS for 1 h at room temperature, and then for approximately 70 h at 4°C. On the second day of processing, tissue was brought to room temperature for 1 h and then rinsed in 2% NGS. Sections were then incubated for approximately 5 h in the secondary antibody (Jackson Immunoresearch; goat anti-rabbit Cy2) diluted 1:300 in 2% NGS.

**Figure 1:**
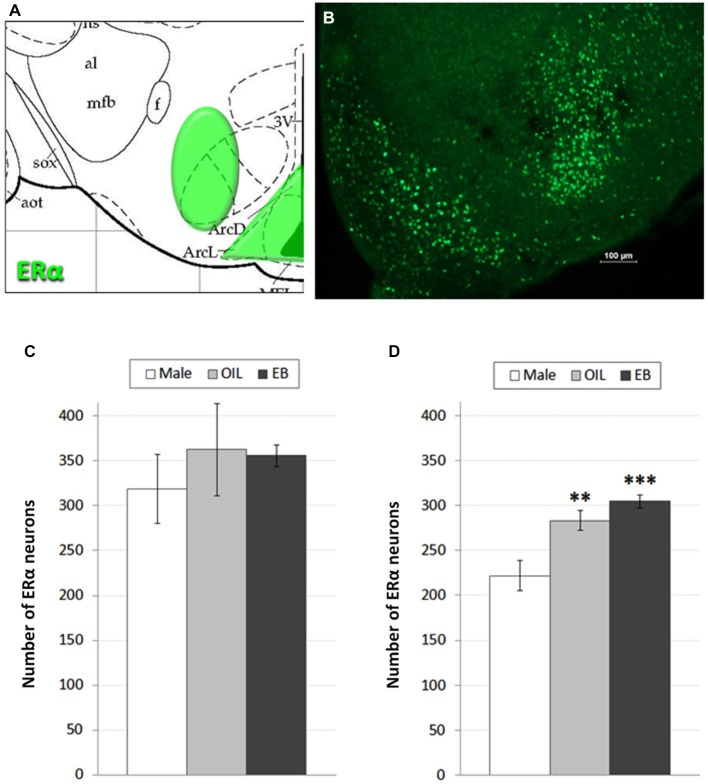
**ERα neurons in the hypothalamus. (A)** Atlas drawing (adapted from Paxinos and Watson, 1998) showing approximate location of ERα-containing neurons (green shading) in the Arcuate nucleus (ARC) of the hypothalamus and the Ventromedial hypothalamus (VMH); **(B)** digital photomicrograph (scale bar = 100 μm) of ERα-immunolabeled neurons in the ARC and VMH. Graphs showing mean numbers of ERα-immunolabeled neurons in **(C)** the VMH and **(D)** ARC of male rats (white bars; *n* = 5–6), and of ovariectomized (OVX) rats given the oil vehicle (OIL; gray bars; *n* = 5–6) or estradiol benzoate (EB; black bars; *n* = 7). **significantly greater than Male (*p* < 0.01). ***significantly greater than Male (*p* < 0.001).

Despite targeted elimination of ERα in the VMH, rats nonetheless decrease feeding in response to estradiol treatment (Musatov et al., 2007), suggesting that additional populations of ERα are involved in the control of feeding. Accordingly, in addition to assessment of ERα in the VMH, we also assessed the possibility of HSD2-ERα associations in the adjacent ARC, which also has been implicated in the control of feeding (Woods and Stricker, 2008). ERα-immunoreactivity was visualized in 2–3 representative sections from the ARC and VMH of each rat based on neuroanatomical landmarks and coordinates as described by Paxinos and Watson (Paxinos and Watson, 1998) (−2.30 to −3.30 mm relative to bregma; see Figure 1A). These sections were matched between subjects and, with exposure time held constant, digital photomicrographs from one side of each section were taken and saved for later quantification of ERα-immunoreactive neurons. Counts from each area were averaged for each rat, and group means for each area (Male: *n* = 5; OIL: *n* = 5–6; EB: *n* = 7) were calculated from the averaged counts.

#### HSD2 Immunolabeling

As described in our previous publication (Fan et al., 2010), sections were incubated in the primary antibody (SantaCruz; rabbit anti-HSD2)^2^ diluted 1:500 in 2% NGS for 1 h at room temperature, and then for approximately 48 h at 4°C. On the second day of processing, tissue was brought to room temperature for 1 h, rinsed in 2% NGS, and then incubated for approximately 5 h in the secondary antibody (Jackson Immunoresearch; goat anti-rabbit Cy3) diluted 1:200 in 2% NGS.

**Figure 2:**
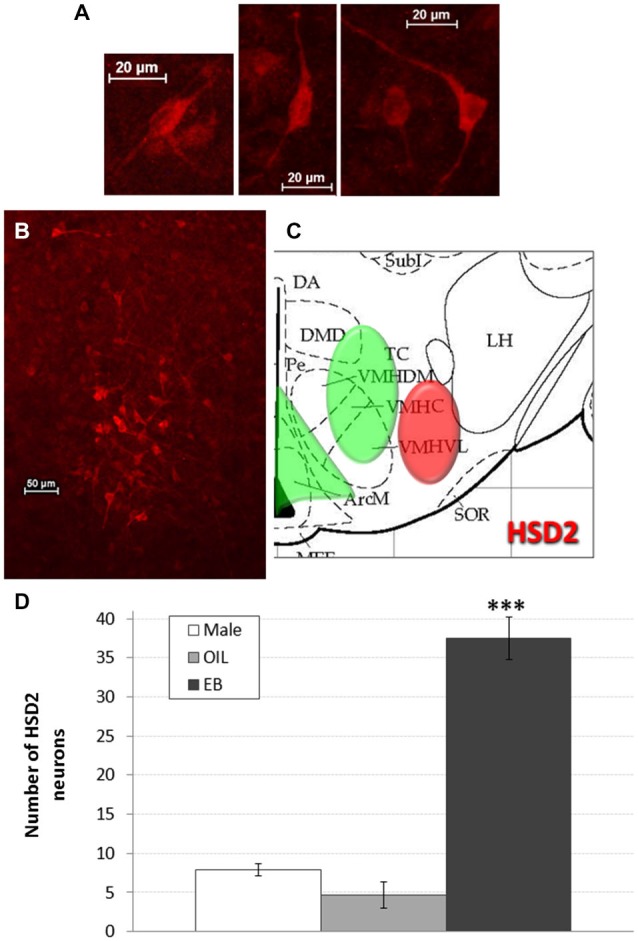
**HSD2 neurons in the hypothalamus. (A)** High magnification digital photomicrographs (scale bars = 20 μm) showing the morphology of HSD2-immunolabeled neurons in the hypothalamus; **(B)** digital photomicrograph (scale bar = 50 μm) of HSD2-immunolabeled neurons in the hypothalamus of an OVX rat given estradiol-benzoate and **(C)** atlas drawing (adapted from Paxinos and Watson, 1998) showing approximate location of HSD2-containing neurons (red shading) and ERα-containing cells (green shading) in the hypothalamus. **(D)** Graph showing mean number of HSD2-immunolabeled neurons in the hypothalamus of male rats (white bars; *n* = 5), and of OVX rats given oil vehicle (OIL; gray bars; *n* = 3) or estradiol benzoate (EB; black bars; *n* = 7). ***significantly greater than Male and OIL (both *p*s < 0.001).

HSD2-immunoreactivity was visualized in the hypothalamus at the approximate level of the ventromedial nucleus (VMN). Putative HSD2-immunoreactive neurons were identified based on morphology (intense, cytoplasmic staining with prominent projections; see Figure 2A) and, with exposure time held constant, digital photomicrographs were taken from one side in each of 2–3 representative sections that were matched between subjects. These micrographs were saved for later quantification of HSD2 neurons. Counts were averaged for each rat, and group means (Male: *n* = 5; OIL: *n* = 3; EB: *n* = 7) were calculated from the averaged counts.

#### HSD2 Double-Immunolabeling

After HSD2-immunolabeling, tissue from subsets of these rats was rinsed in 2% NGS and then processed to identify additional neuronal phenotypes. Given that HSD2-labeling was sparse in both male and OIL-treated OVX rats (Figure 2D), we focused on double-immunolabeling in EB-treated OVX rats. It should be noted that the primary antibodies for both ERα and HSD2 were raised in rabbit, thereby precluding double-immunolabeling for ERα and HSD2 using these techniques.

#### HSD2 and Tyrosine Hydroxylase (TH)

After HSD2-immunolabeling, forebrain sections from EB-treated rats (*n* = 6) were processed to visualize catecholaminergic neurons and fibers in the hypothalamus at the level of the VMH by immunolabeling for tyrosine hydroxylase (TH), the rate limiting enzyme in catecholamine biosynthesis. Sections were incubated in the primary antibody (Millipore; mouse anti-TH; MM_NF-MAB318)^3^ diluted 1:5000 in 2% NGS for 1 h at room temperature, and then for approximately 48 h at 4°C. On the second day of processing, tissue was brought to room temperature for 1 h, rinsed in 2% NGS, and then incubated for approximately 5 h in the secondary antibody (Jackson Immunoresearch; goat anti-mouse Cy2) diluted 1:300 in 2% NGS.

#### HSD2 and Oxytocin (OT)

After HSD2-immunolabeling, forebrain sections from EB-treated rats (*n* = 5) were processed to visualize oxytocinergic neurons and fibers in the hypothalamus at the level of the VMH and extending rostrally to the paraventricular nucleus (PVN) and supraoptic nucleus (SON) of the hypothalamus (−2.12 to −0.80 mm relative to bregma; see Figure 4A). Sections were incubated in the primary antibody (Chemicon; mouse anti-OT; MM_NF-MAB5296)^4^ diluted 1:25000 in 2% NGS for 1 h at room temperature, and then for approximately 48 h at 4°C. On the second day of processing, tissue was brought to room temperature for 1 h, rinsed in 2% NGS and then incubated for 5 h in the secondary antibody (Jackson Immunoresearch: goat anti-mouse Cy2) diluted 1:300 in 2% NGS.

**Figure 3:**
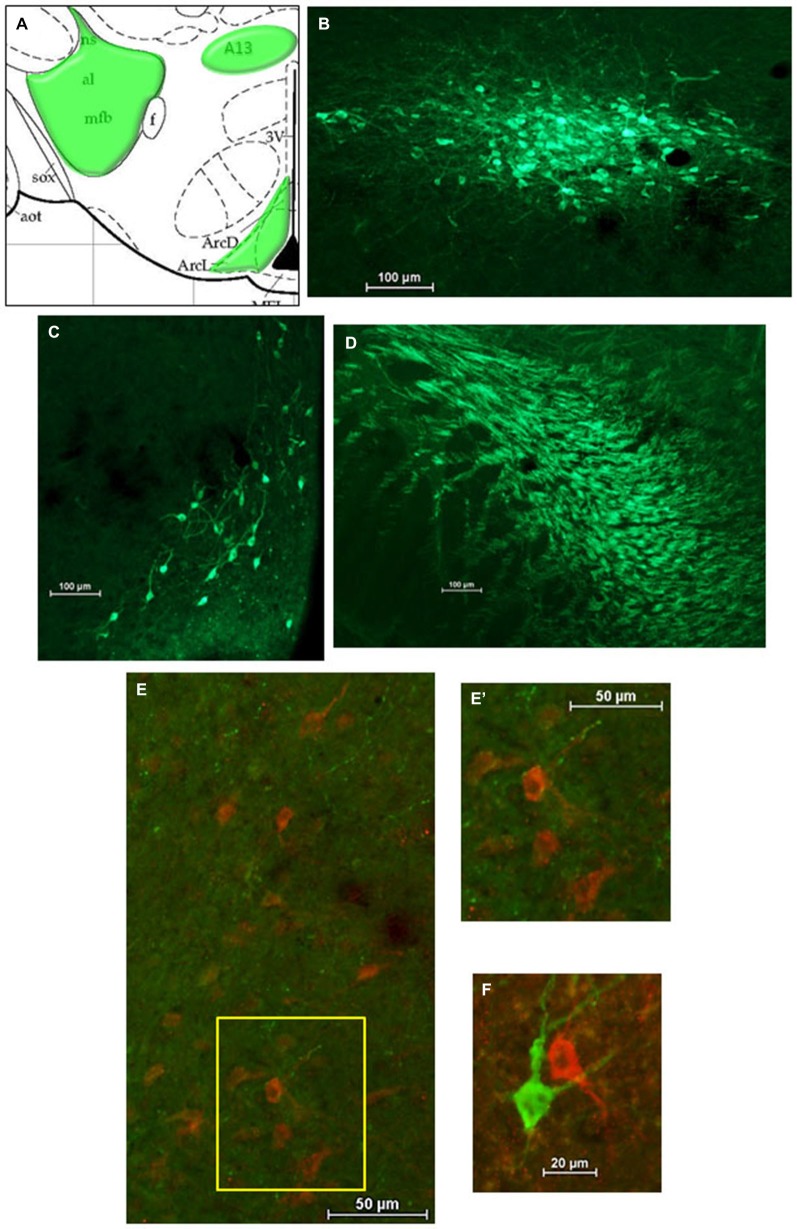
**Catecholamines in the hypothalamus. (A)** Atlas drawing (adapted from Paxinos and Watson, 1998) showing approximate locations of catecholamine neurons and fibers (green shading) in the hypothalamus. Digital photomicrographs (scale bars = 100 μm) of tyrosine hydroxylase (TH)-immunolabeled neurons in **(B)** the A13 region of the hypothalamus and **(C)** the Arcuate Nucleus (ARC), and TH-immunolabeled fibers **(D)** in the medial forebrain bundle (MFB). Merged digital photomicrographs (scale bars = 50 μm, 20 μm) of immunolabeling for HSD2 (red) and TH (TH; green). **(E)** TH-immunolabeled fibers within a population of HSD2-immunolabeled neurons. Area in box on left is shown at higher magnification on the right **(E’). (F)** HSD2- and TH-immunolabeled neurons in close proximity.

**Figure 4:**
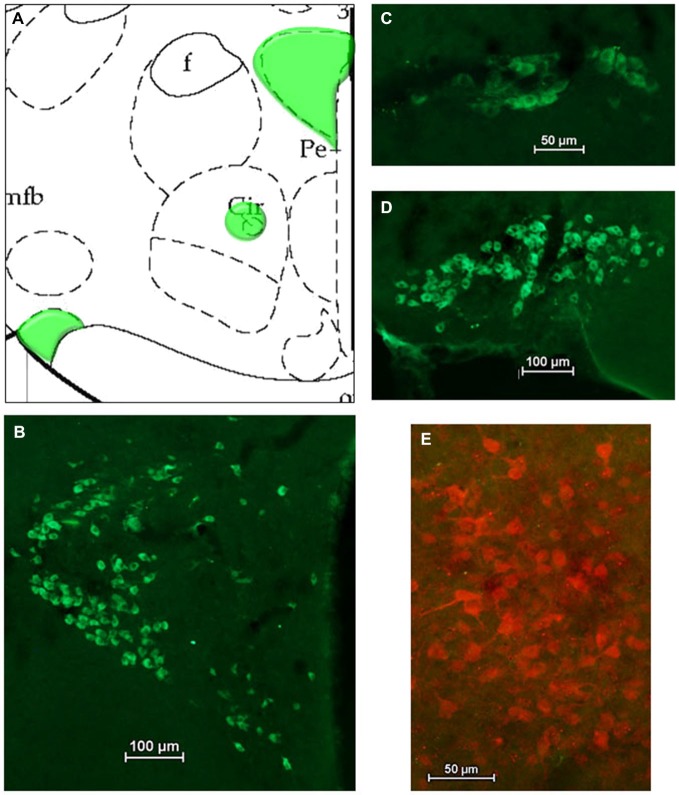
**Oxytocin (OT) in the hypothalamus. (A)** Atlas drawing (adapted from Paxinos and Watson, 1998) showing approximate locations of OT neurons (green shading) in the hypothalamus. Digital photomicrographs (scale bars = 50 μm, 100 um) of OT immunolabeled neurons in **(B)** the Paraventricular nucleus (PVN), **(C)** the Nucleus Circularis, **(D)** the Supraoptic nucleus (SON). **(E)** Merged digital photomicrograph (scale bar = 50 μm) of immunolabeling for HSD2 (red) and OT (green).

For analyses of double-immunolabeling, NIS Elements software was used to overlay fluorescent images. HSD2-labeling appeared as bright red cytoplasmic labeling in putative HSD2 neurons (Figures 2A,B); TH- or OT-labeling appeared as bright green labeling in catecholaminergic (Figures 3B–D) or oxytocinergic neurons or fibers (Figures 4B–D). In merged images, therefore, both green and red labeling could be observed (e.g., Figures 3E,E’,F).

### Statistical Analysis

All values are shown as group means ± standard errors of the mean. Uterine weights were analyzed using a 2-tailed *t*-test. Percent change in body weight, numbers of ERα-immunolabeled neurons in the VMH, numbers of ERα-immunolabeled neurons in the ARC, and numbers of HSD2-immunolabeled neurons each were analyzed using 1-way Analysis of Variance (ANOVA) with group (EB, OIL, MALE) as a factor. Pairwise comparisons of statistically significant effects were further examined using Fisher’s LSD tests.

## Results

### Body Weight and Uterine Weight

Body weight of the three groups on Day 1 and Day 4 are presented in Table 1, along with the percent change from Day 1 to Day 4. As expected (Wade, 1972; Blaustein and Wade, 1976; Roy and Wade, 1977; Geary and Asarian, 1999; Krause et al., 2006; Graves et al., 2011), EB-treated rats lost weight during the 4-day protocol. One-way ANOVA indicated a significant effect of group on the percent change in body weight (*F*_2,13_ = 70.832, *p* < 0.001), and pairwise comparisons revealed that the percent change in EB-treated OVX rats was significantly less than that after oil injections in male rats and in OVX rats (both *p*s < 0.001). Although male rats and OIL-treated OVX rats gained weight during the protocol, the percent change was significantly greater (*p* < 0.01) in male rats.

**Table 1:** **Body weight and uterine weight**.

		Male	OIL	EB
Body weight (g)	Day 1	367.7 ± 12.5	263.5 ± 8.4	278.7 ± 9.5
	Day 4	396.0 ± 13.5	272.5 ± 28.5	268.9 ± 9.5
	% change	7.71 ± 0.43	3.44 ± 0.73^1^	−3.55 ± 0.53^2^
Uterine weight (mg/10 cm)			39.5 ± 8.0	93.6 ± 9.2^3^

*Body weight and body weight change in ovariectomized (OVX) rats given oil vehicle (OIL; *n* = 6) or estradiol benzoate (EB; *n* = 7) and in male rats given oil vehicle (Male; *n* = 3) during the 4-day protocol. Body weight of Male rats that were not given oil vehicle are not included in Table 1; however, on Day 4, body weight of all males included in this study (*n* = 5) was 422.8 ± 8.5 g. Uterine weights were determined in a subset of OVX rats at sacrifice (OIL: *n* = 4, EB: *n* = 5). ^1^significantly less than Male (*p* < 0.01); ^2^significantly less than Male and OIL (both *p*s < 0.001); ^3^significantly greater than OIL (*p* < 0.001)*.

EB treatment also had the expected effect on uterine weight (Graves et al., 2011). As shown in Table 1, uterine weight in EB-treated OVX rats was significantly greater (*p* < 0.01) than that in OIL-treated OVX rats.

### Immunolabeling

#### ERα

Figures 1A,B shows a schematic of the approximate location of ERα-labeled cells in the VMH and ARC, along with a representative digital photomicrograph of ERα-immunolabeling in these areas. Mean numbers of ERα-labeled cells in the VMH and ARC are given in Figures 1C,D. One-way ANOVA indicated no effect of group on the number of ERα-labeled cells in the VMH. In contrast, numbers of ERα-labeled cells in the ARC depended on group (*F*_2,15_ = 13.884, *p* < 0.001). Pairwise comparisons revealed greater numbers of ERα-labeled cells in both EB-treated (*p* < 0.001) and OIL-treated (*p* < 0.01) OVX rats compared to those in Male rats, but no differences between EB- and OIL-treated rats.

#### HSD2

Figures 2B,C shows a representative digital photomicrograph of the cluster of HSD2-immunolabeled neurons in the hypothalamus of an EB-treated OVX rat, along with a schematic of their approximate location. The cluster of HSD2-labeled neurons was anatomically segregated from and lateral to the populations of ERα-labeled cells in both the ARC and the VMH, as indicated on the schematic. Mean numbers of HSD2-labeled neurons are given in Figure 2D. One-way ANOVA indicated a significant effect of group on the number of HSD2-labeled neurons (*F*_2,12_ = 63.285, *p* < 0.001), and pairwise comparisons revealed that the number of HSD2-labeled neurons in EB-treated OVX rats (37.5 ± 2.7 neurons) was significantly greater than those in OIL-treated OVX and male rats (both *p*s < 0.001). In fact, there were few HSD2-labeled neurons in either male rats or OIL-treated OVX rats (less than eight and five neurons, respectively), and the numbers did not differ between these two groups.

#### HSD2 and TH

Figure 3A shows a schematic of the approximate location of TH-labeled neurons and fibers in several areas of the hypothalamus at the level of the VMN. Also shown are representative digital photomicrographs (Figures 3B–D) of TH-immunolabeled neurons in the A13 catecholamine cell group and in the ARC, as well as TH-labeled fibers of the medial forebrain bundle (MFB). Although TH-immunolabeled neurons in the ARC and the A13 region display prominent projections, they did not appear to extend into the cluster of HSD2-labeled neurons. Similarly, the large aggregate of TH-labeled fibers which comprise the MFB did not appear to project to HSD2-labeled neurons. Nonetheless, merged digital photomicrographs of TH- and HSD2-immunolabeling showed a sparse incursion of TH-labeled fibers into the area of HSD2-labeled neurons, some of which appeared to contact HSD2-labeled neurons (Figures 3E,E’). In a few isolated cases, neurons labeled for TH or for HSD2 were observed in close proximity, though clearly were different neurons (Figure 3F).

#### HSD2 and OT

Figures 4A–D shows a schematic of the approximate location of OT-labeled neurons in several areas of the hypothalamus, along with representative digital photomicrographs of OT-immunolabeled neurons in the PVN, Nucleus Circularis, and SON. In general, these neurons did not display conspicuous projections, though punctate OT-labeling was observed in the median eminence, as expected (not shown). Nonetheless, there did not appear to be OT projections to the cluster of HSD2-labeled neurons, as evidenced by the merged digital photomicrograph of OT- and HSD2-immunolabeling (Figure 4E).

## Discussion

Based on previous reports of estradiol-mediated changes in the number of HSD2-containing neurons in the NTS (Fan et al., 2010), the presence of HSD2-containing neurons in the VMH (Geerling et al., 2006b), and the involvement of ERα in the VMH in the regulation of food intake (Musatov et al., 2007), we hypothesized that HSD2-containing neurons in the hypothalamus contribute to centrally-mediated control of feeding by direct interactions with ERα or by indirect interactions with other neurotransmitters previously shown to play a role in feeding. Thus, in this initial approach, we opted to use immunohistochemical methods and focused on neuroanatomical associations between HSD2 and ERα, OT, and catecholamines. Our rationale in these studies rested on the hypothesized co-segregation of HSD2 and ERα in the VMH, which clearly was not found. However, an important caveat to this rationale is that, despite targeted elimination of ERα in the VMH, rats nonetheless decrease feeding in response to estradiol treatment (Musatov et al., 2007), suggesting that additional populations of ERα are involved in the control of feeding. With this in mind, we included the adjacent ARC, which also has been implicated in feeding (Woods and Stricker, 2008), in our assessment of HSD2-ERα associations.

The results were unexpected on several levels. First, HSD2-containing cells with a clear neuronal profile were located as an aggregate in more lateral regions of the hypothalamus. HSD2 immunolabeling previously was reported to occur in the VMH (Geerling et al., 2006b), and we also observed more widespread nuclear HSD2-labeling in the medial parts of the hypothalamus, particularly in close proximity to the third ventricle. However, the HSD2-immunolabled cells that exhibited a distinct neuronal morphology were concentrated in the lateral hypothalamus, and, surprisingly, were observed primarily in EB-treated OVX rats. It is possible that different antibodies employed differentially recognize slightly different epitopes with discrete cellular localizations. Alternatively, our investigation of estrogen-mediated sex differences in this neuronal phenotype may have allowed the detection of this more laterally located population. In either case, we opted to focus on this aggregate of HSD2-containing neurons, which were anatomically segregated from the ERα-immunolabeled cells in both the VMH and the ARC. Thus, if these HSD2-containing neurons interact with ERα to decrease feeding, it is not via co-localization with populations of ERα-containing neurons located in either the VMH or the ARC.

Nonetheless, the numbers of HSD2-containing neurons were profoundly influenced by estradiol, a finding similar to our observations of estradiol-induced effects on HSD2-containing neurons in the hindbrain (Fan et al., 2010). It is unclear at present what mechanism underlies this effect in either area. There was scattered ERα immunolabeling in more lateral areas of the hypothalamus in addition to the dense populations in the VMH and ARC, so the striking increase in the number of HSD2-containing neurons in the more lateral hypothalamus may be attributable to an ERα-mediated increase in gene expression in this population of neurons. Alternatively, the β subtype of ERs may be critical in increasing the number of HSD2-containing neurons, nor can we rule out effects attributable to the g-protein coupled ERs. Finally, “upstream” changes in a neuronal circuit—potentially one that includes neurons in the VMH or ARC—may impact this population of HSD2-containing neurons.

We saw no differences in the number of ERα in the VMH between the two groups of OVX rats, nor between OVX rats and male rats. Given the well-known suppression of feeding by estradiol (Wade, 1972; Blaustein and Wade, 1976; Roy and Wade, 1977; Geary and Asarian, 1999; Krause et al., 2006), and previous findings of hyperphagia and body weight gain when ERα in the VMH are depleted (Musatov et al., 2007), along with reports that 72-h fasting decreases ERα immunoreactivity in the VMH of OVX rats (Jones and Wade, 2002), we expected that food intake by these groups would be related to the number of ERα in the VMH. Clearly, that expectation was not met. It is possible that ERα in the VMH do not underlie differences in feeding by rats under baseline conditions. Instead, their influence may be revealed only when the entire population is eliminated, and food intake and body weight increase to extreme levels. Even then, however, rats appear to retain some level of responsiveness to estradiol (Musatov et al., 2007), which points to a complex interplay among central populations of ERα. Consistent with this idea, we observed sex differences in the number of ERα in the ARC, another important central area in the regulation of feeding (Woods and Stricker, 2008), and in which fasting has been reported to decrease ERα immunoreactivity (Jones and Wade, 2002). However, under *ad libitum* feeding conditions used in our study, the difference in ERα numbers was sex- but not estrogen-dependent and, presumably, was not related to feeding. Thus, an alternative, and not mutually exclusive, possibility is that the *sensitivity* of ERα is the critical factor in the effect of estradiol to inhibit feeding, rather than the *number* of ERα present in a particular area (or areas). On the other hand, there may have been differences in the time course of changes in ERα expression that we could not detect with our 4-day EB-replacement protocol. Clearly, additional studies with a broader array of pharmacological and molecular biological approaches will be necessary to better understand the role of these populations of ERα in the control of feeding.

The observation that HSD2-containing neurons do not co-segregate with ERα in the VMH or the ARC does not in itself negate our hypothesis of a role for HSD2 in the central control of food intake. Rather, the preferential increase in numbers of these neurons by estradiol may allow modulation of a hypothalamic circuit involved in feeding via convergence of input to HSD2-containing neurons. Accordingly, we also examined input to HSD2-containing neurons from neurotransmitters that are known to play a role in the control of feeding. OT inhibition of food intake requires release of OT within the CNS and subsequent activation of central OT receptors (e.g., Olson et al., 1991). Using immunohistochemical methods, we saw no evidence of OT fibers in the vicinity of HSD2-containing neurons, whereas OT projections were apparent in the median eminence. On the surface, these findings might seem to suggest that OT input to HSD2 neurons is not associated with the inhibition of feeding. However, mismatch between projections and receptors have been reported for the OT system in other parts of the CNS (see Sabatier et al., 2013 for review). Thus, the possibility of volume transmission of OT to activate receptors on HSD2-containing neurons cannot be ruled out, particularly since OT binding in the VMH has been reported to increase with administration of estrogen (Tribollet et al., 1990; Arsenijevic et al., 1995). Additional studies will be necessary to determine more conclusively whether OT and HSD2 interact in the hypothalamus.

In terms of catecholamines, noradrenaline signaling in the hypothalamus has been shown to contribute to hyperphagia via interactions with leptin and/or NPY (see Wellman, 2000 for review); however, the possibility of dopaminergic signaling associated with the rewarding aspects of food intake also exists (Palmiter, 2007). Merged images revealed close apposition of TH-immunolabled fibers with HSD2 neurons in EB-treated rats that could indicate synaptic contacts. TH-immunolabeled fibers in the vicinity of HSD2-containing neurons were sparse; nonetheless, these findings suggest the possibility of interactions between catecholamines and HSD2 in the control of feeding. However, more detailed analysis using, e.g., confocal microscopy will be necessary to determine more conclusively whether these appositions are, in fact, synaptic contacts. In short, additional studies will be necessary to determine whether TH-immunolabled fibers do, in fact, synapse on HSD2-labeling neurons. If so, subsequent studies may focus on identifying which catecholamine, the receptor mechanism by which HSD2 neuronal responses may be affected and, most important for our hypothesis, the influence of such responses on food intake.

In summary, we observed striking effects of estradiol treatment on the number of HSD2-containing neurons in the more lateral hypothalamus, but no evidence for a direct associations between hypothalamic HSD2 and ERα or HSD2 and OT. Neuroanatomical associations between hypothalamic HSD2 and catecholamines are modest and subject to verification of synaptic contacts. Thus, these studies, which began with the hypothesis that HSD2-containing neurons in the hypothalamus contribute to the control of feeding via associations with ERα or other neurotransmitters, did not conclusively identify a candidate neuroanatomical substrate that would support this hypothesis. Nonetheless, some possibilities have been identified that bear further investigation. At the same time, it is clear that subsequent studies using pharmacological and/or molecular biology methods will be necessary to determine whether these HSD2-containing neurons do, in fact, influence feeding under conditions of elevated estradiol and whether catecholamines are involved.

We end with the acknowledgment that despite our hypothesis of a role for hypothalamic HSD2-containing neurons in the control of feeding, and preliminary evidence from these pilot studies, other possibilities remain. First, given that HSD2 is a signature of aldosterone-sensitive tissues, HSD2 neurons in the lateral hypothalamus may serve as a target for aldosterone in the forebrain, and activity in this hypothalamic population may be related to body sodium homeostasis, as it is in the hindbrain (Geerling et al., 2006a; Geerling and Loewy, 2007). An intriguing alternative, given the role of HSD2 in the interplay between glucocorticoids and mineralocorticoids (Náray-Fejes-Tóth et al., 1998), is that the hypothalamic population of HSD2-containing neurons alters stress responsiveness in the presence of estradiol (e.g., Solomon and Herman, 2009). Finally, the estradiol sensitivity of this population and the known role of the VMH in sexual behavior (e.g., Flanagan-Cato, 2011) raise the possibility that hypothalamic HSD2-containing neurons may contribute to the control of reproductive behaviors. Clearly, further investigations are necessary to understand the functional role of HSD2-containing neurons in the hypothalamus, as well as the implications of the estradiol-mediated changes in the numbers of these neurons in the hypothalamus and in the hindbrain.

## Conflict of Interest Statement

The authors declare that the research was conducted in the absence of any commercial or financial relationships that could be construed as a potential conflict of interest.

## Footnotes

^1^http://www.emdmillipore.com/US/en/product/Anti-Estrogen-Receptor-%CE%B1-Antibody,MM_NF-06-935#anchor_COA; NG1838275

^2^http://datasheets.scbt.com/sc-20176.pdf

^3^http://www.emdmillipore.com/US/en/product/Anti-Tyrosine-Hydroxylase-Antibody,MM_NF-MAB318#anchor_COA; LV1594176

^4^http://www.emdmillipore.com/US/en/product/Anti-Oxytocin-Antibody%2C-clone-4G11,MM_NF-MAB5296#anchor_COA; 1979939

## References

[B1] Anand B. K. Brobeck J. R. (1951). Localization of a “feeding center” in the hypothalamus of the rat. Proc. Soc. Expe. Biol. Med. 2, 323–324. 10.3181/00379727-77-18766 14854036

[B2] Arsenijevic Y. Dreifess J. J. Vallet P. Marguerat A. Tribollet E. (1995). Reduced binding of oxytocin in the rat brain during aging. Brain Res. 698, 275–279. 10.1016/0006-8993(95)01020-v 8581497

[B3] Blaustein J. D. Wade G. N. (1976). Ovarian influences on the meal patterns of female rats. Physiol. Behav. 17, 201–208. 10.1016/0031-9384(76)90064-0 1033580

[B6] Eckel L. A. Houpt T. A. Geary N. (2000). Spontaneous meal patterns in female rats with and without access to running wheels. Physiol. Behav. 70, 397–405. 10.1016/s0031-9384(00)00278-x 11006440

[B7] Fan L. Smith C. E. Curtis K. S. (2010). Regional differences in estradiol effects on numbers of HSD2-containing neurons in the nucleus of the solitary tract of rats. Brain Res. 1358, 89–101. 10.1016/j.brainres.2010.08.037 20728435 PMC2949458

[B8] Flanagan-Cato L. M. (2011). Sex differences in the neural circuit that mediates female sexual receptivity. Front. Neuroendocrinol. 32, 124–136. 10.1016/j.yfrne.2011.02.008 21338620 PMC3085563

[B9] Geary N. Asarian L. (1999). Cyclic estradiol treatment normalizes body weight and test meal size in ovariectomized rats. Physiol. Behav. 67, 141–147. 10.1016/s0031-9384(99)00060-8 10463640

[B11] Geerling J. C. Engeland W. C. Kawata M. Loewy A. D. (2006a). Aldosterone target neurons in the Nucleus Tractus Solitarius drive sodium appetite. J. Neurosci. 26, 411–417. 10.1523/jneurosci.3115-05.2006 16407537 PMC6674421

[B12] Geerling J. C. Kawata M. Loewy A. D. (2006b). Aldosterone-sensitive neurons in the rat central nervous system. J. Comp. Neurol. 494, 515–527. 10.1002/cne.20808 16320254

[B10] Geerling J. C. Loewy A. D. (2007). Sodium depletion activates the aldosterone-sensitive neurons in the NTS independently of thirst. Amer. J. Phys. Reg. Integrat. Comp. Physiol. 292, R1338–R1348. 10.1152/ajpregu.00391.2006 17068161

[B13] Graves N. S. Hayes H. Fan L. Curtis K. S. (2011). Time course of behavioral, physiological and morphological changes after estradiol treatment of ovariectomized rats. Physiol. Behav. 103, 261–267. 10.1016/j.physbeh.2011.02.017 21324332 PMC3476457

[B14] Hetherington A. W. Ranson S. W. (1940). Hypothalamic lesions and adiposity in the rat. Anat. Record 78, 149–172. 10.1002/ar.1090780203

[B42] Hetherington A. W. Ranson S. W. (1942). The relation of various hypothalamic lesions to adiposity in the rat. J. Comp. Neurol. 76, 475–499. 10.1002/cne.900760308

[B15] Jones J. E. Wade G. N. (2002). Acute fasting decreases sexual receptivity and neural estrogen receptor-alpha in female rats. Physiol. Behav. 77, 19–25. 10.1016/s0031-9384(02)00780-1 12213498

[B16] Kisley L. R. Sakai R. R. Ma L. Y. Fluharty S. J. (1999). Ovarian steroid regulation of angiotensin II-induced water intake in the rat. Am. J. Physiol. 276, R90–R96. 9887181 10.1152/ajpregu.1999.276.1.R90

[B17] Krause E. G. Curtis K. S. Stincic T. L. Markle J. P. Contreras R. J. (2006). Oestrogen and weight loss decrease isoproterenol-induced Fos immunoreactivity and angiotensin type 1 mRNA in the subfornical organ of female rats. J. Physiol. 573, 251–262. 10.1113/jphysiol.2006.106740 16543266 PMC1779697

[B18] Laflamme N. Nappi R. E. Drolet G. Labrie C. Rivest S. (1998). Expression and neuropeptidergic characterization of estrogen receptors (ERalpha and ERbeta) throughout the rat brain: anatomical evidence of distinct roles of each subtype. J. Neurobiol. 36, 357–378. 10.1002/(SICI)1097-4695(19980905)36:3%3C357::AID-NEU5%3E3.0.CO;2-V 9733072

[B19] Marino M. Galluzzo P. Ascenzi P. (2006). Estrogen signal multiple pathways to impact gene transcription. Curr. Genomics 7, 497–508. 10.2174/138920206779315737 18369406 PMC2269003

[B20] Musatov S. Chen W. Pfaff D. W. Mobbs C. V. Yang X. J. Clegg D. J.. (2007). Silencing of estrogen receptor alpha in the ventromedial nucleus of hypothalamus leads to metabolic syndrome. Proc. Natl. Acad. Sci. U S A 104, 2501–2506. 10.1073/pnas.0610787104 17284595 PMC1892990

[B21] Náray-Fejes-Tóth A. Colombowala I. K. Fejes-Tóth G. (1998). The role of 11beta-hydroxysteroid dehydrogenase in steroid hormone specificity. J. Steroid Biochem. Mol. Biol. 65, 311–316. 10.1016/S0960-0760(98)00009-0 9699885

[B22] Olson B. R. Drutarosky M. D. Chow M. S. Hruby V. J. Stricker E. M. Verbalis J. G. (1991). Oxytocin and an oxytocin agonist administered centrally decrease food intake in rats. Peptides 12, 113–118. 10.1016/0196-9781(91)90176-p 1646995

[B23] Olszewski P. K. Klockars A. Schiöth H. B. Levine A. S. (2010). Oxytocin as feeding inhibitor: maintaining homeostasis in consummatory behavior. Pharmacol. Biochem. Behav. 97, 47–54. 10.1016/j.pbb.2010.05.026 20595062 PMC2952698

[B24] Palmiter R. D. (2007). Is dopamine a physiologically relevant mediator of feeding behavior? Trends Neurosci. 30, 375–381. 10.1016/j.tins.2007.06.004 17604133

[B25] Paxinos G. Watson C. (1998). The Rat Brain in Stereotaxic Coordinates. 4th Edn. San Diego: Academic Press.

[B26] Roesch D. M. (2006). Effects of selective estrogen receptor agonists on food intake and body weight gain in rats. Physiol. Behav. 87, 39–44. 10.1016/j.physbeh.2005.08.035 16181647

[B27] Roy E. J. Wade G. N. (1977). Role of food intake in estradiol-induced body weight changes in female rats. Horm. Behav. 8, 265–274. 10.1016/0018-506x(77)90001-0 881167

[B28] Sabatier N. Leng G. Menzies J. (2013). Oxytocin, feeding and satiety. Front Endocrinol. 4:35. 10.3389/fendo.2013.00035 23518828 PMC3603288

[B29] Santollo J. Katzenellenbogen B. S. Katzenellenbogen J. A. Eckel L. A. (2010). Activation of ER[alpha] is necessary for estradiol’s anorexigenic effect in female rats. Horm. Behav. 58, 872–877. 10.1016/j.yhbeh.2010.08.012 20807534 PMC2982904

[B30] Santollo J. Wiley M. D. Eckel L. A. (2007). Acute activation of ER alpha decreases food intake, meal size and body weight in ovariectomized rats. Am. J. Physiol. Regul. Integr. Comp. Physiol. 293, R2194–R2201. 10.1152/ajpregu.00385.2007 17942491

[B31] Schwartz M. W. Woods S. C. Porte D. Jr. Seeley R. J. Baskin D. G. (2000). Central nervous system control of food intake. Nature 404,661–671. 10.1038/35007534 10766253

[B32] Shughrue P. J. Lane M. V. Merchenthaler I. (1997). Comparative distribution of estrogen receptor-alpha and -beta mRNA in the rat central nervous system. J. Comp. Neurol. 388, 507–525. 10.1002/(SICI)1096-9861(19971201)388:4%3C507::AID-CNE1%3E3.0.CO;2-6 9388012

[B33] Simerly R. B. Chang C. Muramatsu M. Swanson L. W. (1990). Distribution of androgen and estrogen receptor mRNA-containing cells in the rat brain: an in situ hybridization study. J. Comp. Neurol. 294, 76–95. 10.1002/cne.902940107 2324335

[B34] Solomon M. B. Herman J. P. (2009). Sex differences in psychopathology: Of gonads, adrenals and mental illness. Physiol. Behav. 97, 250–258. 10.1016/j.physbeh.2009.02.033 19275906 PMC4539024

[B35] Tribollet E. Augidier S. Dubois-Dauphin M. Dreifuss J. J. (1990). Gonadal steroids regulate oxytocin receptors in the brain of male and female rats. An autoradiographical study. Brain Res. 511, 129–140. 10.1016/0006-8993(90)90232-z 2158853

[B43] Uchoa E. T. Mendes da Silva L. E. C. de Castro M. Antunes-Rodrigues J. Elias L. L. K. (2009). Hypothalamic oxytocin neurons modulate hypophagic effect induced by adrenalectomy. Horm. Behav. 56, 532–538. 10.1016/j.yhbeh.2009.09.007 19778539

[B44] Verbalis J. G. Blackburn R. E. Olson B. R. Stricker E. M. (1993). Central oxytocin inhibition of food and salt ingestion: a mechanism for intake regulation of solute homeostasis. Regul. Pept. 45, 149–154. 10.1016/0167-0115(93)90198-h 8511338

[B36] Wade G. N. (1972). Gonadal hormones and behavioral regulation of body weight. Physiol. Behav. 8, 523–534. 10.1016/0031-9384(72)90340-x 4556652

[B37] Watson R. E. Jr. Wiegand S. J. Clough R. W. Hoffman G. E. (1986). Use of cryoprotectant to maintain long-term peptide immunoreactivity and tissue morphology. Peptides 7, 155–159. 10.1016/0196-9781(86)90076-8 3520509

[B38] Wellman P. J. (2000). Norepinephrine and the control of food intake. Nutrition 16, 837–842. 10.1016/s0899-9007(00)00415-9 11054588

[B39] Woods S. C. D’Alessio D. A. (2008). Central control of body weight and appetite. J. Clin. Endocrinol. Metab. 93, S37–S50. 10.1210/jc.2008-1630 18987269 PMC2585760

[B40] Woods S. C. Stricker E. M. eds. (2008). Food Intake and Metabolism. 3rd Edn. San Diego: Academic Press.

[B41] Woolley C. S. McEwen B. S. (1993). Roles of estradiol and progesterone in regulation of hippocampal dendritic spine density during the estrous cycle in the rat. J. Comp. Neurol. 336, 293–306. 10.1002/cne.903360210 8245220

